# Penetration enhancement effects of topically applied crosslinked hyaluronic acid

**DOI:** 10.1111/ics.70076

**Published:** 2026-01-19

**Authors:** Aaron Cohen, Raphael Legouffe, Krystal House, Paul Duhamel, Aurore Tomezyk, Mathieu Gaudin, Junhong Mao, David Bonnel

**Affiliations:** ^1^ Personal Care Department Colgate‐Palmolive Company Piscataway New Jersey USA; ^2^ Aliri Loos France; ^3^ Cross Category Research & Innovation Department Colgate‐Palmolive Company Piscataway New Jersey USA

**Keywords:** delivery, hyaluronic acid, MALDI‐MSI, polymers, skin barrier, topical delivery

## Abstract

**Objective:**

In this manuscript, we report that crosslinked hyaluronic acid (HA), a skincare active with well‐documented hydration and skin protection benefits, can act as a penetration enhancer for the topical delivery of skincare actives. High‐performance liquid chromatography (HPLC) and matrix‐assisted laser desorption/ionization mass spectrometry imaging (MALDI‐MSI) studies revealed that crosslinked HA can boost the topical delivery of both small‐molecule actives and macromolecules alike.

**Methods:**

Porcine ear skin and human skin explants were used for the HPLC and MALDI‐MSI studies, respectively. Topical delivery studies were conducted with simple cosmetic vehicles containing either the active of interest or the active of interest plus crosslinked HA. Delivery studies were conducted for 24 h, after which skin explants were analysed to measure the level of topically delivered active.

**Results:**

HPLC analysis revealed that crosslinked HA selectively boosted the topical delivery of hydrophilic actives, which we attributed to the strong water‐binding properties of crosslinked HA. MALDI‐MSI revealed that crosslinked HA could also boost the topical delivery of a medium‐molecular‐weight HA species (~200–400 kDa), demonstrating that the penetrating enhancing properties of crosslinked HA benefit both small molecules and macromolecules alike.

**Conclusions:**

Crosslinked HA can boost the topical delivery of relevant skincare actives that range in size from small‐molecule actives (<500 Da) to large macromolecules (>10 000 Da) like linear HA itself. Combined with its well‐established and previously documented skin benefits, this makes crosslinked HA a highly versatile ingredient for topical skincare products.

## Graphical abstract

Cross‐linked hyaluronic acid (HA) was demonstrated to enhance the topical delivery of a variety of hydrophilic actives into the skin. This phenomenon was observed for both small molecules and macromolecules, the latter of which was analysed with MALDI mass spectrometry imaging (MALDI‐MSI).

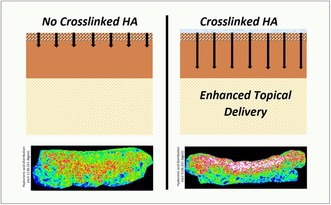

## Résumé

**Objectif:**

Dans ce manuscrit, nous rapportons que l’acide hyaluronique (AH) réticulé, un produit de soin de la peau actif avec des bénéfices bien documentés en matière d’hydratation et de protection de la peau, peut agir comme un amplificateur de pénétration pour l’administration topique des produits de soin de la peau actifs. Les études de chromatographie en phase liquide à haute performance (HPLC, *high‐performance liquid chromatography*) et d’imagerie par spectrométrie de masse à désorption/ionisation laser assistée par matrice (MALDI‐MSI, *matrix‐assisted laser desorption/ionization mass spectrometry imaging*) ont révélé que l’AH réticulé peut stimuler l’administration topique des actifs, qu’il s’agisse de petites molécules ou de macromolécules.

**Méthodes:**

Des explants de peau d’oreille de porc et de peau humaine ont été utilisés pour les études d’HPLC et de MALDI‐MSI, respectivement. Des études d’administration topique ont été menées avec des excipients cosmétiques simples contenant soit la substance active d’intérêt, soit la substance active d’intérêt plus l’AH réticulé. Des études d’administration ont été menées pendant 24 heures, après quoi les explants de peau ont été analysés afin de mesurer le taux d’agent actif administré par voie topique.

**Résultats:**

L’analyse de l’HPLC a révélé que l’AH réticulé stimulait de manière sélective l’administration topique d’actifs hydrophiles, ce que nous avons attribué à ses puissantes propriétés de liaison à l’eau. La MALDI‐MSI a révélé que l’AH réticulé pouvait également stimuler l’administration topique d’une espèce d’AH de poids moléculaire moyen (~200 à 400 kDa), démontrant que les propriétés pénétrantes d’amélioration de l’AH réticulé bénéficient tant aux petites molécules et qu’aux macromolécules.

**Conclusions:**

L’AH réticulé peut stimuler l’administration topique d’actifs cutanés pertinents de taille variable, allant des petites molécules actives (<500 Da) aux grandes macromolécules (>10 000 Da), comme l’AH linéaire lui‐même. Associé à ses bienfaits bien établis et précédemment documentés sur la peau, l’AH réticulé est un ingrédient très polyvalent pour les produits de soins de la peau topiques.

## INTRODUCTION

Hyaluronic acid (HA) is a linear glycosaminoglycan (GAG) consisting of a repeat unit of glucuronic acid and *N*‐acetylglucosamine [1, 2]. HA has long been used in topical cosmetic formulations [3, 4], where it can impart a myriad of benefits including an exceptional ability to moisturize the skin [5, 6], anti‐aging benefits [7, 8, 9] and skin barrier repair [10, 11]. HA provides a wide range of benefits because it is not a single material per se, but a family of molecules that can vary greatly in molecular weight [12, 13, 14]. High molecular weight HA, with a molecular weight typically above 1 MDa, will have negligible skin penetration but instead remains on the surface of the skin, providing a rich depot of hydration [15, 16]. On the other hand, low molecular weight HA, with a molecular weight typically below 100 kDa, can exhibit significant penetration into the skin, where it can impart wound healing and anti‐aging benefits, along with potential anti‐inflammatory properties [17, 18, 19].

Beyond the aforementioned benefits, there is an extensive body of work highlighting the role HA can play in boosting topical delivery [20, 21, 22, 23]. As a general trend, lower molecular weight HA species typically provide greater topical delivery benefits compared with higher molecular weight HA species [24, 25, 26]. Manconi et al. used an HA species of about 200–400 kDa to prepare HA‐based vesicles (hyalurosomes), which could boost the topical delivery of curcumin [24], while Jung and Lim et al. prepared a nanohydrogel from a low molecular weight HA (20 kDa) derivative for the transdermal delivery of indocyanine green [25]. In a 2015 paper, Witting et al. investigated hydrogels prepared from HAs of three molecular weights (5 kDa, 100 kDa, 1 MDa) and showed that the lowest molecular weight species could preferentially boost the topical delivery of bovine serum albumin (BSA) [26]. The authors hypothesize that this is caused by the increased ability of the lowest molecular weight HA to penetrate into the skin, where it can co‐transport the BSA.

An attractive form of HA in cosmetic products is crosslinked HA, which is generally produced by reacting linear HA with a polyfunctional cross‐linking agent [27, 28, 29]. Cross‐linking individual HA chains generates a network structure that effectively has an infinite molecular weight, imparting this material with significantly higher structural integrity compared to any form of non‐crosslinked HA [30, 31, 32]. These unique properties grant crosslinked HA superior hydration benefits compared to its non‐crosslinked counterpart [33, 34]. Furthermore, the network‐like structure of crosslinked HA allows it to form a film on the surface of the skin, where it can provide wound healing benefits [35, 36, 37, 38].

To date, there is only minimal work exploring the delivery benefits of crosslinked HA. It is hypothesized that HA operates as a topical delivery agent by hydrating and ultimately increasing the permeability of the stratum corneum [20]. Therefore, because of its previously mentioned benefits, crosslinked HA may act as an excellent delivery agent. In a 2015 article, a variant of crosslinked HA containing hydroxyethyl cellulose was shown to aid in the transdermal delivery of the antioxidant isoliquiritigenin [39]. The authors speculate that the modified crosslinked HA can swell the stratum corneum, creating a temporary disruption in the skin's outermost barrier. In a separate example, crosslinked HA nanogels were prepared via a “click chemistry” process and used to modulate the delivery and release of model fluorescent dyes [40]. However, both works only investigated the delivery of a single active and/or model fluorescent dyes. Additionally, both reports employed customized, non‐commercially available crosslinked HA that required multiple synthetic steps to prepare, which could limit its wider scale application. Therefore, a rigorous investigation of the delivery properties of commercially available crosslinked HA would be beneficial to the skincare industry.

In this article, we report that crosslinked HA can act as a powerful penetration enhancer for the topical delivery of a variety of skincare actives. We observe that crosslinked HA can preferentially boost the topical delivery of hydrophilic skincare actives, which we attribute to the water‐binding properties of crosslinked HA. Additionally, through the use of matrix‐assisted laser desorption/ionization mass spectrometry imaging (MALDI‐MSI), we demonstrate that crosslinked HA can also boost the topical delivery of a medium molecular weight HA species, indicating that this phenomenon can be used for both small molecules and macromolecules. This makes crosslinked HA an ideal skincare agent with a wide range of benefits.

## MATERIALS AND METHODS

### Materials

Arginine and α‐tocopherol were obtained from Sigma‐Aldrich (St. Louis, MO). Carnosine, supplied under the trade name Dragosine, was obtained from Symrise (Branchburg, NJ). Sodium PCA (50% w/w aqueous solution) and zinc PCA (neat material) were both obtained from Ajinomoto (Raleigh, NC), as Ajidew N‐50 and Ajidew ZN‐100, respectively. The crosslinked HA used in this study was Hyacross Hyaluronic Acid Elastomer TL100, which was obtained from Bloomage Biotech (Jinan, China). This material is an aqueous gel of roughly 1%–5% w/w active ingredient. The high molecular weight HA used in some of the studies was Hybloom Sodium Hyaluronate (HA‐T, Bloomage Biotech), with a molecular weight between 1000 and 1800 kDa. For all studies, active ingredients (2% w/w carnosine, 1% w/w arginine, 0.5% w/w α‐tocopherol, 2% w/w sodium/zinc PCA, 1% w/w medium molecular weight HA) and crosslinked HA (1%–3% w/w depending on the study) were loaded into a simple topical vehicle prepared in house, which consisted of roughly 90% w/w water, along with isoprene glycol and 1,2‐hexanediol, and thickened with sodium polyacrylate. The pH of this vehicle was 6.15.

For MALDI‐MSI, 2,5‐dihydroxybenzoic acid (2,5 DHB), *N,N*‐dimethylaniline (DMA) and haematoxylin and eosin (H&E) used for staining were purchased from Sigma‐Aldrich (St. Louis, MO, USA). LC‐MS grade methanol (MeOH) and LC‐MS grade water (H_2_O) were obtained from Thermo Fisher Scientific (Germany). Indium tin oxide (ITO) slides were purchased from Delta Technologies (Loveland, CO, USA), and Pertex mounting medium was obtained from VWR (Fontenay‐sous‐Bois, France).

### Skin sample and preparation

Experimental studies analysed via HPLC (delivery of carnosine, α‐tocopherol, arginine, sodium PCA, zinc PCA) were conducted on porcine ear skin (Animal Technologies, Tyler, TX). The porcine ear skin samples were shaved and had the subcutaneous fat layer removed. Experimental studies analysed via MALDI‐MSI were conducted on frozen human skin (Genoskin, Salem, MA). The samples were obtained from the abdomen and tested for the absence of hepatitis and HIV prior to use.

All studies used circular pieces of skin with a diameter of roughly 3.5 cm. Skin samples were visually inspected to ensure that no breaks and/or disruptions in the skin barrier were observed. The appropriate formulation was applied to the skin surface and rubbed for 30 s to ensure that the final mass of sample applied was 50 ± 2 mg. Once all samples were prepared, skin explants were placed on a DHC‐6T transdermal system (Logan Instrument Corp, Somerset, NJ). The receiver fluid, which consisted of deionized water, was held at a constant temperature of 32°C. Skin samples were clamped and the penetration study was conducted for 24 h before analysis.

### Skin analysis and HPLC methodology

For samples treated with carnosine, arginine, sodium PCA or zinc PCA, the surface of the skin was first cleaned with a Kimwipe to remove residual topical formulation. Then, the first 20 layers of the skin were removed using tape strips (D100 D‐Squame Standard Sampling Discs, Clinical & Derm) which were subsequently discarded. The remaining skin was then cut into small pieces and extracted with 15 mL of solvent (DI water for arginine, sodium PCA and zinc PCA; 60/40 v/v acetonitrile/water for carnosine). Analyte was then extracted from the skin homogenate through a combination of sonication and vortexing.

For samples treated with α‐tocopherol, the surface of the skin was first cleaned with a Kimwipe to remove residual topical formulation. Then, the first three layers of the skin were removed using tape strips. The remainder of the skin was placed into a PBS solution heated to 60°C for 1 min, after which the epidermis could be removed from the dermis using an X‐acto knife. Analyte was extracted from all three regions in either 95/5 v/v acetonitrile/water (tape strips) or methanol (epidermis and dermis) through a combination of sonication and vortexing. Separation of the epidermis and dermis was only performed for samples treated with water insoluble α‐tocopherol; other samples were treated with water soluble actives and thus separating the layers in a PBS solution would lead to substantial loss of the active material. Receptor fluid was not analysed in any of the studies; previous work (unpublished) has shown that under these experimental conditions no measurable level of analyte will permeate through the skin and into the receptor fluid. Aside from the MALDI‐MSI study, all delivery studies were repeated to ensure reproducibility.

#### HPLC derivatization of arginine

Because arginine possesses minimal inherent UV‐absorbance, it underwent a derivatization reaction prior to HPLC analysis with 4‐Fluoro‐7‐nitrobenzofurazan (NBD‐F) following scientific literature [41]. In brief, to a centrifuge tube was added 1 mL of the arginine sample, 1 mL of an NBD‐F solution in water (concentration ~0.2 mg/mL) and 2 mL of an aqueous buffer solution (0.1 M KH_2_PO_4_ and 0.02 M EDTA, with the pH adjusted to ~9.0 with NaOH). The samples were then reacted at 60°C for 30 min in an incubator with a speed of 150 rpm. After the incubation, samples were removed and placed in an ice bath, followed by the addition of 1 mL of 0.2 M HCl to quench the reaction. Then 0.4 mL of this reaction was added to a scintillation vial, along with another 1.6 mL of acetonitrile and gently mixed. This final mixture was filtered into an HPLC vial for analysis.

#### HPLC derivatization of sodium PCA and zinc PCA

Because sodium PCA and zinc PCA possess minimal inherent UV‐absorbance, they were both derivatized prior to HPLC analysis with 2,4′‐dibromoacetophenone, following scientific literature [42]. In brief, to a centrifuge tube was added 0.5 mL of the sodium/zinc PCA sample, 0.5 mL of a 10 mg/mL acetonitrile solution of 2,4′‐dibromoacetophenone and 0.5 mL of a 5 mg/mL acetonitrile solution of triethanolamine. The samples were then reacted at 60°C in an incubator with a speed of 200 rpm for either 3 h (sodium PCA) or 18 h (zinc PCA). After the reaction, the samples were left to cool, and then another 1.5 mL of acetonitrile was added, after which the samples were filtered into HPLC vials.

#### HPLC detection of α‐tocopherol

Details of this method can be found in Table 1.

**TABLE 1:** Overview of HPLC methodologies for detection of carnosine, arginine, α‐tocopherol (vitamin E) and sodium PCA/zinc PCA.

	Carnosine	Arginine^a^	α‐Tocopherol (vitamin E)	Sodium PCA/zinc PCA^a^, ^b^
Instrument	Agilent 1200 LC	Agilent 1200 LC	Agilent 1200 LC	Agilent 1260 LC
Column	Agilent Zorbax NH2 (PN 880952–708)	Agilent Poroshell 120 HILIC‐Z (PN 683975–924)	Agilent Zorbax RX‐C18 (PN 883967–902)	Agilent Zorbax RX‐C18 (PN 883967–902)
Mobile phase	60/40 v/v acetonitrile /aqueous buffer	90/10 v/v acetonitrile /20 mM NH_4_HCO_2_ (pH 3.0)	99/1 v/v methanol/ water	Gradient (see below)
Injection volume	20 μL	20 μL	80 μL	5 μL
Flow rate	1.0 mL/min	1.0 mL/min	1.0 mL/min	0.8 mL/min
Detection wavelength	210 nm	470 nm	292 nm	260 nm
Retention time	18.0 min	7.0 min	10.0 min	16.5 min

^a^ Sample underwent pre‐column derivatization.

^b^ Only anionic component (pyroglutamate) was analysed.

#### HPLC detection of carnosine

Carnosine was detected using a previously reported method [43], the details of which are seen in Table 1.

#### HPLC detection of derivatized arginine

Details for the HPLC methodology of derivatized arginine can be found in Table 1.

#### HPLC detection of derivatized sodium PCA and zinc PCA

Details for the HPLC analysis of derivatized sodium PCA and zinc PCA can be found in Table 1. The following gradient was used, with A = water and B = acetonitrile: hold at 90/10 v/v A/B 0 to 5 min, 90/10 v/v A/B to 60/40 v/v A/B 5 to 15 min, 60/40 v/v A/B to 0/100 v/v A/B 15 to 25 min, hold at 0/100 v/v A/B 25 to 29 min, 0/100 v/v A/B to 90/10 v/v A/B 29 to 33 min, hold at 90/10 v/v A/B 33 to 37 min.

### Atomic force microscopy (AFM)

Topography imaging of linear and crosslinked HA films was performed on a SmartSPM (Horiba Scientific). The HA solutions were drop‐cast on freshly‐cleaved muscovite mica and allowed to dry under ambient conditions. Mica is a common AFM substrate [44] and was used here as a model for human skin, both having a negative surface charge [45, 46]. Images were acquired in tapping mode in air at room temperature with an ARROW‐UHFAuD (NanoWorld) probe with a nominal resonance frequency of *f* = 2000 kHz. The scan rate for all images was 1.0 Hz with 512 × 512 resolution. AFM images were processed using Gwyddion 2.66 SPM analysis software [47].

### MALDI‐MSI

#### Tissue section preparation for MALDI imaging

Prior to analysis, treated human skin samples were first cleaned with a Kimwipe to remove residual sample on the skin surface. Human skin samples stored at −80°C were placed inside a cryostat (HM560—Thermo Fisher Scientific, Germany). The cryostat temperature was maintained between −20 and −25°C. Each tissue sample was fixed onto the cryostat support using a few millilitres of optimum cutting temperature gel (OCT; Qpath freeze gel, VWR, Fontenay‐sous‐Bois, France). For each sample, tissue sections of 10 μm thickness were prepared and collected onto indium tin oxide (ITO) slides (Delta Technologies, Loveland, CO, USA) for MALDI‐MSI analysis. Additional adjacent sections were collected onto Superfrost slides (Thermo Fisher Scientific, Germany) for H&E staining. Each slide was cryo‐dried inside the cryostat for 15 min. If not analysed immediately, slides were stored at −80°C for future use.

Before spraying the MALDI matrix, enzymatic digestion on the tissue was performed as previously described [48]. Enzyme H1136 from Sigma Aldrich (St. Louis, MO, USA) was selected at a concentration of 300 Units/mL and prepared in a specific buffer (20 mM sodium phosphate with 77 mM sodium chloride and 0.01% w/v Bovine Serum Albumin, pH 7.0 at room temperature). Enzyme solution (500 μL) was sprayed onto tissue sections using the TM‐sprayer™ system (HTX Imaging, Chapel Hill, NC, USA), followed by an 18‐h incubation at 37°C before MALDI analysis.

#### MALDI matrix preparation, deposition and acquisition

2,5‐DHB‐DMA solution was prepared at 40 mg/mL in methanol/water/*N,N*‐dimethylaniline (1:1:0.02 v:v:v). The MALDI matrix solutions were sprayed using the HTX TM‐sprayer system (HTX Imaging, Chapel Hill, NC, USA) over tissue sections, following the same method and preparation across all analyses.

MALDI images were obtained using a 7T MALDI‐FTICR (Solarix, Bruker Daltonics, Bremen, Germany) equipped with a SmartBeam II laser operating at a repetition rate of 2000 Hz in negative ion mode. Mass spectra were acquired in CASI (Continuous Accumulation of Selected Ion) mode within the 1000–1200 Da mass range, with a spatial resolution of 80 μm. Each mass spectrum has been recorded following 300 consecutive laser shots per image position. The mass spectrometer and imaging parameters were controlled using FTMS Control 2.2.0 and FlexImaging 5.0 software (Bruker Daltonics, Bremen, Germany).

#### Histological staining

Haematoxylin and eosin (H&E) staining was performed on tissue sections after MALDI matrix removal on ITO slides, as well as on adjacent sections mounted on Superfrost slides. The H&E staining was used to correlate regions of interest (epidermis and dermis) with molecular distributions of HA obtained via MALDI‐MSI. The epidermis was delineated based on histological features, specifically the visualization of the stratum corneum and basal layer. For the dermis, the first third under the epidermis was classified as the upper dermis, while the last two‐thirds were considered the lower dermis. High‐resolution images of the stained tissue sections were generated using the S60 scanner (Hamamatsu, Hamamatsu City, Shizuoka Pref., Japan) at 20× magnification.

#### Depth profiling

To determine the penetration profiles of HA, a Region of Interest (ROI) was delineated per tissue section based on its molecular distribution in Multimaging™ software (Aliri, Loos, France). The ROI included the epidermis, upper dermis and lower dermis. For each ROI, the intensity of HA per position (pixel) was extracted from the dataset. An alignment of the pixels along the *x*‐axis was performed using Multimaging™ software, with the 0‐mm depth value defined as the first pixel line obtained from the epidermis. From this alignment, a new matrix of intensity values per position was created.

### Statistical analysis

Means, standard deviations and *p*‐values (two‐tailed *t*‐test) were calculated using Microsoft Excel for all studies.

## RESULTS

### Intradermal delivery of carnosine, arginine and α‐tocopherol

**FIGURE 1:**
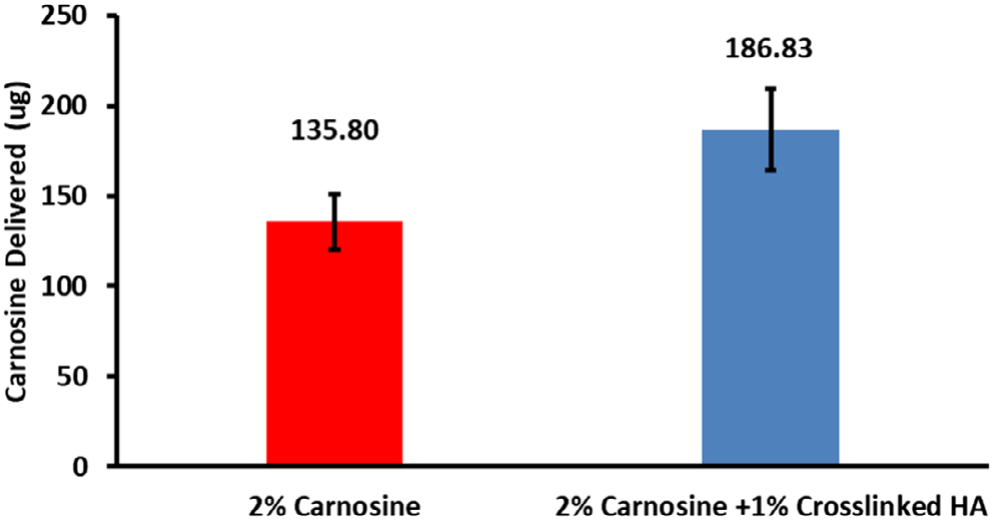
Effect of crosslinked HA on the topical delivery of carnosine. *N* = 4 per sample, 136 ± 15 μg for samples with 2% w/w carnosine versus 187 ± 22 μg of delivered active for samples with 2% w/w carnosine and 1% w/w crosslinked HA, *p* < 0.02.

**FIGURE 2:**
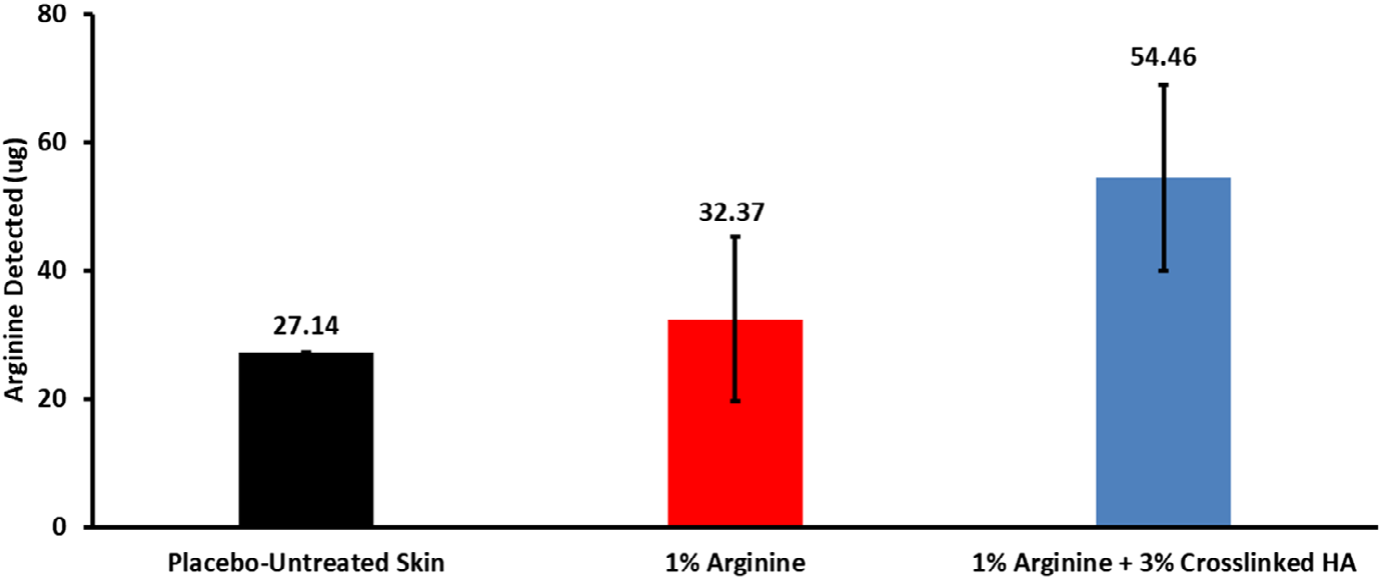
Effect of crosslinked HA on the topical delivery of arginine. 27 μg endogenous arginine in untreated, 32 ± 13 μg of total detected arginine with formula containing 1% w/w arginine (*n* = 4), 54 ± 14 μg of total detected arginine with formula containing 1% w/w arginine and 3% w/w crosslinked HA (*n* = 4), *p* ~ 0.06.

**FIGURE 3:**
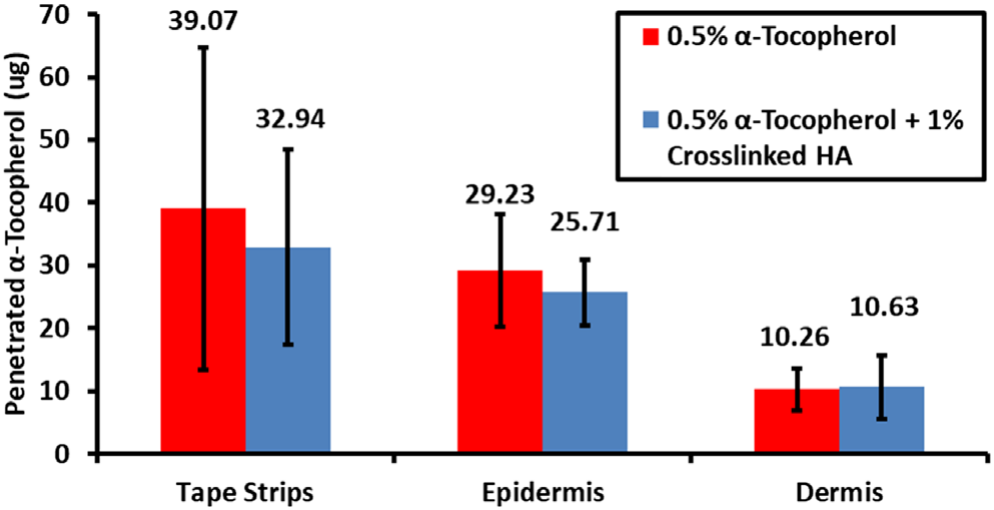
Effect of crosslinked HA on the topical delivery of α‐tocopherol (vitamin E). *N* = 4 per sample. Tape Strips: 39 ± 26 μg for 0.5% w/w α‐tocopherol, 33 ± 16 μg for 0.5% w/w α‐tocopherol and 1% w/w crosslinked HA, *p* ~ 0.35. Epidermis: 29 ± 9 μg for 0.5% w/w α‐tocopherol, 26 ± 5 μg for 0.5% w/w α‐tocopherol and 1% w/w crosslinked HA, *p* ~ 0.26. Dermis: 10 ± 3 μg for 0.5% w/w tocopherol, 11 ± 5 μg for 0.5% w/w α‐tocopherol and 1% w/w crosslinked HA, *p* ~ 0.45.

The topical delivery of carnosine, arginine and α‐tocopherol after 24 h are shown in Figures 1, 2, 3 respectively and show the effect (or lack thereof) of the addition of crosslinked HA on active penetration into the skin. Porcine ear skin contains significant levels of endogenous arginine, and therefore untreated skin was also analysed for this study to account for any background levels of the active (porcine skin also contains extremely small levels of endogenous carnosine and tocopherol, however in our measurements such amounts were so low as to be effectively negligible). For both carnosine and arginine, the first 20 tape strips of the skin were removed and discarded and the remainder of the skin was analysed for penetrated active. In both cases, there is a clear increase in the level of topically delivered active in the underlying tissue. For samples loaded with 2% w/w carnosine, the level of topically delivered active increased by about 70% with the addition of 1% w/w crosslinked HA.

The effect of crosslinked HA on the topical delivery of 1% w/w arginine is seen in Figure 2. Like carnosine, the addition of crosslinked HA causes an increase in the overall level of detected arginine. The effect of crosslinked HA on arginine was slightly more muted in comparison to carnosine. A 3% w/w level of crosslinked HA was needed to boost the topical delivery of arginine compared to 1% w/w crosslinked HA for carnosine. Initial attempts to boost the topical delivery of arginine with only 1% w/w crosslinked HA did not lead to any overall increase (see Figure S1).

The effect of crosslinked HA on the topical delivery of 0.5% w/w α‐tocopherol is seen in Figure 3. For treated skin samples, the first three tape strip layers were removed, and then the remainder of the epidermis was separated from the dermis. As clearly seen in the graph, the addition of 1% w/w crosslinked HA led to no difference in the topical delivery of α‐tocopherol into the skin.

### Effect of non‐crosslinked HA on topical delivery

**FIGURE 4:**
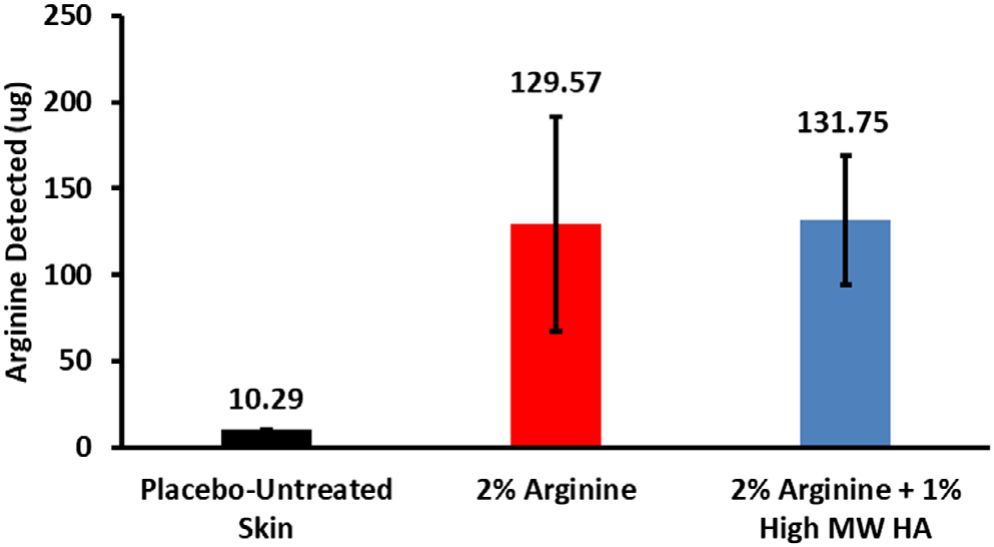
Effect of high molecular weight HA on the topical delivery of arginine. 10 μg endogenous arginine in untreated sample, 130 ± 62 μg of total detected arginine with formula containing 2% w/w arginine (*n* = 4), 132 ± 38 μg of total detected arginine with formula containing 2% w/w arginine and 1% w/w high molecular weight HA (*n* = 4), *p* ~ 0.95.

**FIGURE 5:**
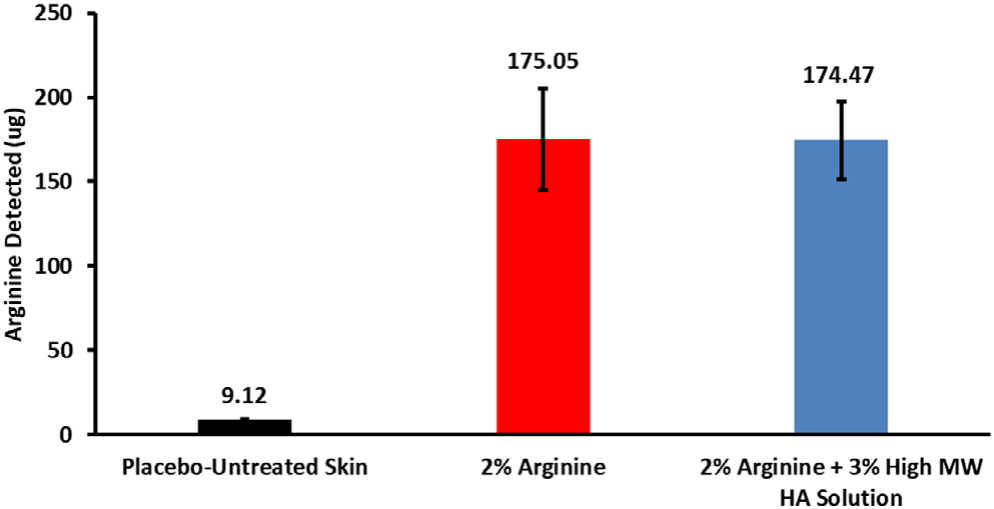
Effect of an aqueous solution of high molecular weight HA on the topical delivery of arginine. 9 μg endogenous arginine in untreated sample, 175 ± 30 μg of total detected arginine with formula containing 2% w/w arginine (*n* = 4), 174 ± 23 μg of total detected arginine with formula containing 2% w/w arginine and 3% w/w solution of high molecular weight HA (*n* = 4), *p* ~ 0.97.

If crosslinked HA's strong hydrating properties are responsible for its topical delivery benefits, then a non‐crosslinked, high molecular weight HA should also act as a potent penetration enhancer, as this form of HA also possesses excellent hydrating properties. This was tested in two studies measuring the topical delivery of 2% w/w arginine, both using a form of HA with a molecular weight of ~1.0 MDa–1.8 MDa. In the first study (Figure 4), HA was directly added into the formula at 1% w/w. In the second study (Figure 5), 3% w/w of an aqueous solution consisting of 1% w/w HA was added into the formula, for a final HA level of 0.03% w/w. In both cases, it is clearly evident that the use of high molecular weight HA has no impact on the topical delivery of arginine, in contrast to 3% w/w crosslinked HA (Figure 2).

### AFM analysis of crosslinked and non‐crosslinked HA

**FIGURE 6:**
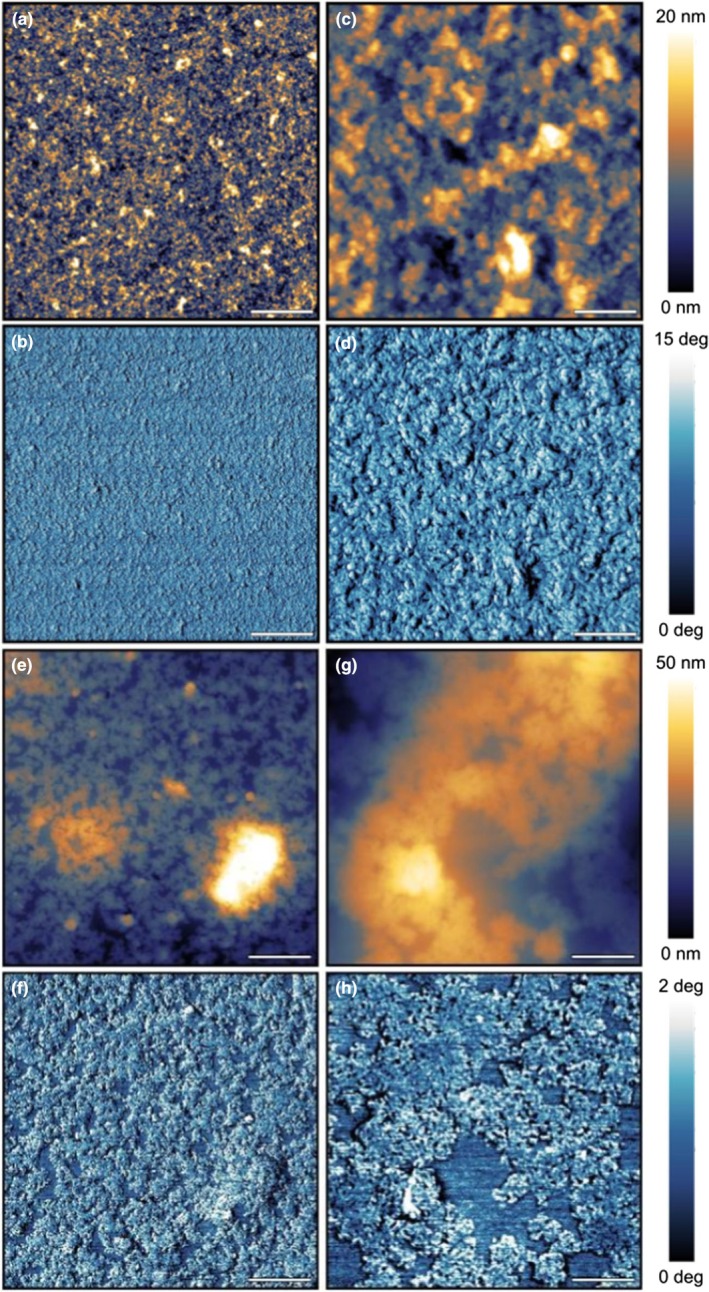
Atomic force microscopy (AFM) images of crosslinked (a–d) and high molecular weight (e–h) HA films on mica. 5 × 5 μm height (a, e) and phase (b, f) images, scale bar is 1 μm. 1 × 1 μm height (c, g) and phase (d, h) images, scale bar is 200 nm. Images have been processed using Gwyiddion where all height images are plane levelled and have a second‐degree polynomial line fitting.

To further explore the differences between high molecular weight HA and crosslinked HA, AFM was performed on samples containing either a 1% w/w aqueous solution of the crosslinked HA or a 1% w/w aqueous solution of the high molecular weight HA. Film formation on mica varies significantly for the high molecular weight and crosslinked HA solutions. The crosslinked HA forms a uniform network structure on the mica surface with the height of the features in the range of 20 nm (Figure 6a–d). The uniformity of the film was confirmed with 50 μm^2^ scans in several areas. In contrast, for the high‐molecular‐weight HA (Figure 6e–h), a heterogeneous surface film is observed with dispersed globular formations of material. Globular formations of HA on mica have been shown previously [49, 50, 51]. The height of these formations is in the range of 50–90 nm while the rest of the surface has an approximately 25 nm heterogeneous covering. More detail of the structure can be seen in the 1 × 1 μm phase images for each film (Figure 6d,h). It is clear that the surface is made up of HA molecules.

### Effect of crosslinked HA on topical delivery of sodium PCA and zinc PCA

**FIGURE 7:**
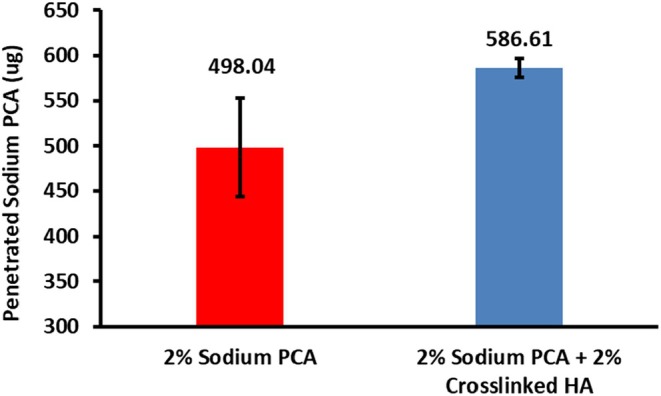
Effect of crosslinked HA on the topical delivery of sodium PCA. *N* = 4 per sample, 498 ± 55 μg for samples with 2% w/w sodium PCA versus 587 ± 10 μg of delivered active for samples with 2% w/w sodium PCA and 2% w/w crosslinked HA, *p* < 0.02.

**FIGURE 8:**
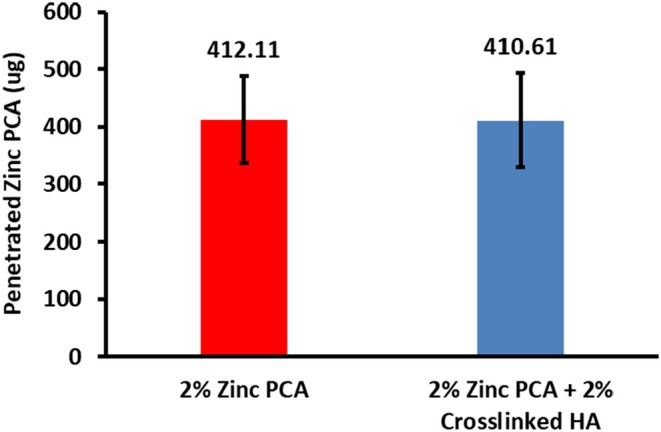
Effect of crosslinked HA on the topical delivery of zinc PCA. *N* = 4 per sample, 412 ± 75 μg for samples with 2% w/w zinc PCA versus 411 ± 82 μg of delivered active for samples with 2% w/w zinc PCA and 2% w/w crosslinked HA, *p* > 0.99.

In a further set of studies, the topical delivery of both sodium PCA and zinc PCA was investigated with and without the addition of crosslinked HA. Sodium PCA is endogenously present as part of the skin's natural moisturizing factor (NMF) [52] and as such, many topical cosmetic formulas contain sodium PCA to provide moisturization benefits [53, 54]. Zinc PCA provides the benefits of PCA along with any potential wound healing/antibacterial benefits afforded by the zinc [55, 56, 57]. The topical delivery of sodium PCA and zinc PCA is displayed in Figures 7 and 8, respectively. Like carnosine and arginine (see above), the first 20 tape strips of the skin were removed and discarded, and the remainder of the skin was analysed for penetrated active. The addition of 2% w/w crosslinked HA enhanced the topical delivery of 2% w/w sodium PCA, as seen in Figure 7. In contrast, the addition of 2% w/w crosslinked HA had no effect on the topical delivery of 2% w/w zinc PCA, as seen in Figure 8 (the skin's endogenous sodium PCA is confined largely to the stratum corneum [52], and as such, no endogenous signals were observed for the deeper regions of the skin that we analysed). Clearly, the presence of Zn^2+^ ions impacts the ability of the crosslinked HA to behave as a penetration enhancer in a manner not observed with the Na^+^ ion.

### MALDI‐MSI of topically delivered linear HA

We also investigated whether crosslinked HA could boost the topical delivery of a macromolecule of interest in the skincare industry, medium molecular weight HA (~200–400 kDa). To analyse the full delivery of HA throughout the skin, MALDI‐MSI was utilized to provide detailed spatial resolution of HA distribution, using a previously reported method [48].

**FIGURE 9:**
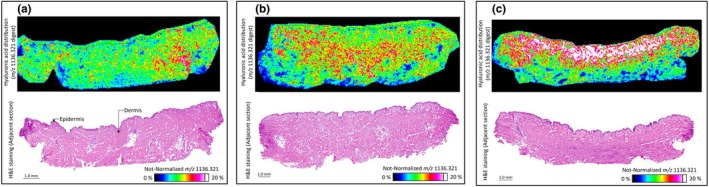
(a–c) MALDI‐MSI of human skin explant treated with placebo formula containing no HA, showing the distribution of endogenous HA in the tissue (a), human skin explant treated with topical formula containing 1% w/w medium molecular weight HA (b) and human skin explant treated with topical formula containing 1% w/w medium molecular weight HA and 3% w/w crosslinked HA.

**FIGURE 10:**
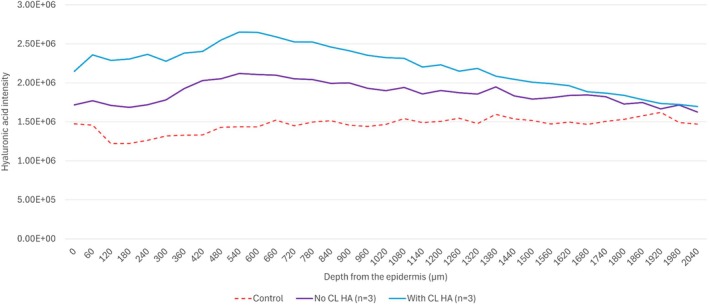
Distribution profile of human skin explants treated with placebo formula (bottom, *N* = 1), topical formula containing 1% w/w medium molecular weight HA (middle, *N* = 3) and topical formula containing 1% w/w medium molecular weight HA and 3% w/w crosslinked HA (top, *N* = 3).

MALDI‐MSI of skin samples treated with placebo formula (no HA), formula with 1% of the medium molecular weight HA species, and 1% w/w of the medium molecular weight HA species and 3% w/w of the crosslinked HA are displayed in Figure 9a–c. Skin samples treated with the placebo reveal a level of endogenous HA throughout the skin that is consistent with previous work (Figure 9a) [48]. Skin samples treated with the topical formula containing 1% w/w medium molecular weight HA (*N* = 3) reveal that some level of this HA can deliver into the skin, even without the inclusion of any kind of delivery vehicle/penetration enhancer (Figure 9b). However, skin samples treated with the topical formula containing 1% w/w medium‐molecular‐weight HA and 3% w/w crosslinked HA (*N* = 3) showed by far the highest level of HA throughout the skin (Figure 9c). The distribution graph of HA detected in the skin (Figure 10) revealed that skin samples treated with the topical formula containing 1% w/w medium molecular weight HA and 3% w/w of the crosslinked HA showed a uniformly higher level of HA throughout the skin compared to skin samples treated with the topical formula containing only 1% w/w medium molecular weight HA.

To confirm that the increased level of HA detected in the skin was indeed a result of more of the medium molecular weight HA penetrating into the skin, and not the result of any of the crosslinked HA penetrating into the skin (the MALDI MSI method cannot distinguish between HA species of different molecular weights and/or cross‐linking), we also analysed skin samples treated with either the placebo topical formula or the topical formula with only 3% w/w of the crosslinked HA and no other form of HA. The results of this study (Figure S2) reveal no major differences in the HA levels for skin samples treated with either sample. This confirms that the differences observed in Figure 9a–c are indeed caused by the increased topical delivery of the medium molecular weight HA, and not by any topical delivery of the crosslinked HA itself. Such results are not surprising, as the effectively infinite molecular weight of the crosslinked HA would prevent it from penetrating into the skin at any meaningful level [29, 58].

## DISCUSSION

Crosslinked HA is a well‐known derivative of HA, with reports of cross‐linking HA and its use as an injectable filler dating back to the 1980s and 1990s [28, 59, 60, 61]. As a topical ingredient, crosslinked HA is known to provide superb hydrating and film forming properties, allowing it to function as a “shield” to block harmful external aggressors [29, 33, 34]. Although most reports on the delivery enhancing properties of HA suggest that lower molecular weights provide superior delivery benefits [24, 25, 26], we hypothesized that crosslinked HA's unique combination of hydrating and film forming properties would enable it to boost the delivery of certain topical ingredients into the skin. Initial investigations revealed that such a property was limited to the topical delivery of hydrophilic actives like carnosine and arginine, and not lipophilic actives like α‐tocopherol (the logP values for carnosine, arginine and α‐tocopherol are predicted as −4 [62], −4.2 [63] and 10.7 [64], respectively). This suggests that the mode of action for crosslinked HA's delivery properties is at least partially related to its ability to create a highly hydrated environment in the upper part of the skin, which would have selective affinity for water‐soluble, hydrophilic actives.

If the mode of action was simply a function of crosslinked HA's hydrating properties, then such an increase in topical delivery would also be observed with non‐crosslinked, high molecular weight HA, as such a form of HA also possesses well‐documented hydrating benefits [5, 65]. The lack of such a delivery benefit for high molecular weight HA (Figures 4 and 5) suggests that other factors are also at play. Atomic force microscopy of high molecular weight HA and crosslinked HA reveals fundamental differences in how they spread and cover a surface. Crosslinked HA forms a relatively homogeneous film over the surface, in contrast to high molecular weight HA, which aggregates in discrete regions. The observed film formation is in agreement with previous reports [35, 36, 37, 38] and helps elucidate crosslinked HA's observed delivery benefits, as film‐forming agents are widely reported to boost the intradermal and transdermal delivery of active materials [66, 67]. Such differences can explain the superior delivery benefits observed with crosslinked HA. This is in keeping with previous literature, which suggests that high molecular weight HA has little to no impact in boosting topical delivery [26].

Sodium and zinc PCA provide a unique opportunity to further probe crosslinked HA's ability to act as a penetration enhancer. Although both share the common pyroglutamate anion (and pyroglutamate was the analyte specifically measured via HPLC), only sodium PCA saw its topical delivery into the skin increased by the addition of crosslinked HA, despite both materials being highly water soluble. In contrast to the sodium ion with a +1 charge, the zinc in zinc PCA has a +2 charge and can typically form complexes with a coordination number of four or six [68, 69, 70]. As an illustration, several reports of zinc hyaluronate describe the ability of the zinc ion to coordinate with several binding sites on the HA chain [71, 72], in contrast to sodium hyaluronate, where the sodium ion only binds to a single site [73]. Because of this, we hypothesized that the zinc ion in zinc PCA could effectively bind to multiple sites of the crosslinked HA, hindering its hydrating and film forming properties and ultimately suppressing its ability to boost the topical delivery of skincare actives.

We also investigated whether crosslinked HA could boost the topical delivery of a medium molecular weight linear HA species (~200–400 kDa). MALDI‐MSI revealed that skin explants treated with a combination of crosslinked HA and medium molecular weight HA (Figure 9c) had a higher level of HA than samples treated with either medium molecular weight HA alone (Figure 9b) or the placebo (to account for endogenous HA in the skin, Figure 9a). Further testing of human skin explants treated with either the placebo or crosslinked HA alone confirmed that the increase in HA level was due to more of the medium molecular weight HA being delivered into the skin, and not from any crosslinked HA absorbing into the skin (see SI). Given its relatively large size (the lowest molecular weight of commercially available HA is approximately ~5–10 kDa), HA has a surprisingly high ability to deliver topically into the skin. Previous studies have indicated that topically applied HA can penetrate into the skin via a transcellular route due to its high affinity for corneocytes [21, 74, 75]. Topical application of HA can lead to swelling of both the corneocytes themselves and the intercellular space between the corneocytes [20, 74]; achieving such a phenomenon through the addition of crosslinked HA likely explains the increased levels of HA observed in skin samples treated with both medium molecular weight HA and crosslinked HA.

The work presented suggests a myriad of future research. Given our proposed mode of action for crosslinked HA to act as a penetration enhancer, other materials that are both highly hydrating and film forming may also provide topical delivery benefits. Among others, many polysaccharide‐based materials are well known to have film forming properties [76, 77, 78], such as chitosan [79, 80] and cellulose derivatives [81, 82]. Polysaccharides in general are also known to have moisturizing properties [83, 84, 85]; given their structural similarities to HA, a more in‐depth study of their active delivery properties would be a potential future direction.

Crosslinked HA's ability to boost the topical delivery of a medium molecular weight HA is also an attractive feature, as the medium molecular weight HA used in this study was about 300 kDa. The topical delivery of macromolecules remains challenging [86, 87], even as the potential to deliver a macromolecule through the skin and avoid oral administration provides many advantages [88, 89]. Further expanding this phenomenon to other macromolecules would be of great interest, particularly as it can bypass more invasive delivery methods of macromolecules such as microneedling [90, 91]. Previous research indicated that the addition of 5% w/v low molecular weight (5 kDa) HA enhanced the topical delivery of bovine serum albumin, a protein with a molecular weight of ~66 kDa [26]. One limitation of that work was the relatively high level of HA used, as 5% neat HA is unlikely to be used in a cosmetic product due to the costs and potential formulation challenges. Therefore, further studies exploring the ability of crosslinked HA to enhance the topical delivery of other macromolecules will be a long‐term research goal of our group.

## CONCLUSIONS

Crosslinked HA was shown to boost the topical delivery of a variety of skincare actives into porcine and human skin explants. Specifically, it can boost the topical delivery of hydrophilic but not lipophilic actives into the skin, a phenomenon we attribute to its strong water binding capacity. In general, we hypothesize that crosslinked HA's ability to enhance topical delivery is due to both its strong hydrating properties and its film forming behaviour, a mode of action that was further explored by AFM analysis. Crosslinked HA can also boost the topical delivery of a medium‐molecular‐weight HA species, as demonstrated by MALDI‐MSI; such a phenomenon can be helpful in boosting the overall benefits of HA present in many cosmetic formulations. Further research by our group will continue to explore other actives whose topical delivery can be enhanced via the incorporation of crosslinked HA as well as investigating other materials with similar topical delivery benefits to crosslinked HA.

## AUTHOR CONTRIBUTIONS

A.C., R.L., K.H. and P.D. performed the research. A.C., R.L., K.H. and J.M. designed the research study. A.T., M.G. and D.B. reviewed the data and ensured quality control. A.C., R.L. and K.H. wrote the paper.

## CONFLICT OF INTEREST STATEMENT

Aaron Cohen and Krystal House are employees of the Colgate‐Palmolive Company. Junhong Mao is a former employee of the Colgate‐Palmolive Company. Raphael Legouffe, Paul Duhamel, Aurore Tomezyk, Mathieu Gaudin and David Bonnel are employees of Aliri.

## ETHICS STATEMENT

This study does not involve any human or animal testing.

## Supporting information


Data S1.

## Data Availability

The data that support the findings of this study are available from the corresponding author upon reasonable request.
