# Supplementary material for: Penetration enhancement effects of topically applied crosslinked hyaluronic acid
Source: Int J Cosmet Sci. 2026 Jan 19;48(4):798–812. doi: 10.1111/ics.70076 (PMC13448370; doi:10.1111/ics.70076)
Supplement: Supplementary file 1 [file ICS-48-798-s001.docx]

*Figure SI1: Effect of 1% Crosslinked HA on Topical Delivery of Arginine*

*Figure SI2: Distribution profile of human skin explants treated with placebo formula and topical formula containing 3% crosslinked HA*
